# SASP Modulation for Cellular Rejuvenation and Tissue Homeostasis: Therapeutic Strategies and Molecular Insights

**DOI:** 10.3390/cells14080608

**Published:** 2025-04-17

**Authors:** Saud Alqahtani, Taha Alqahtani, Krishnaraju Venkatesan, Durgaramani Sivadasan, Rehab Ahmed, Nizar Sirag, Hassabelrasoul Elfadil, Hanem Abdullah Mohamed, Haseena T.A., Rasha Elsayed Ahmed, Pooja Muralidharan, Premalatha Paulsamy

**Affiliations:** 1 Department of Pharmacology, College of Pharmacy, King Khalid University, Abha 62521, Saudi Arabia; 2 Department of Pharmaceutics, College of Pharmacy, Jazan University, Jazan 45142, Saudi Arabia; dsivadasa@jazanu.edu.sa; 3 Division of Microbiology, Immunology and Biotechnology, Department of Natural Products and Alternative Medicine, Faculty of Pharmacy, University of Tabuk, Tabuk 71491, Saudi Arabia; rahmed@ut.edu.sa (R.A.); habdelgadir@ut.edu.sa (H.E.); 4 Department of Natural Products and Alternative Medicine, Faculty of Pharmacy, University of Tabuk, Tabuk 71491, Saudi Arabia; nmona@ut.edu.sa; 5 Pediatric Nursing, College of Nursing, King Khalid University, Abha 62521, Saudi Arabia; haababdullah@kku.edu.sa; 6 Faculty of Nursing, Cairo University, Giza 12613, Egypt; 7 College of Nursing, Mahalah Branch for Girls, King Khalid University, Abha 62521, Saudi Arabia; hkhadar@kku.edu.sa (H.T.); pponnuthai@kku.edu.sa (P.P.); 8 Medical Surgical Nursing, Tanta University, Tanta 31527, Egypt; raelsalem@kku.edu.sa; 9 College of Nursing, King Khalid University, Khamis Mushait 61421, Saudi Arabia; 10 Undergraduate Program, PSG College of Pharmacy, Peelamedu, Coimbatore 641004, India; poojaram45232@gmail.com

**Keywords:** cellular senescence, senescence-associated secretory phenotype (SASP), cellular rejuvenation, aging and tissue homeostasis, SASP modulation strategies

## Abstract

Cellular senescence regulates aging, tissue maintenance, and disease progression through the Senescence-Associated Secretory Phenotype (SASP), a secretory profile of cytokines, chemokines, growth factors, and matrix-remodeling enzymes. While transient SASP aids wound healing, its chronic activation drives inflammation, fibrosis, and tumorigenesis. This review examines SASP’s molecular regulation, dual roles in health and pathology, and therapeutic potential. The following two main strategies are explored: senescence clearance, which eliminates SASP-producing cells, and SASP modulation, which refines secretion to suppress inflammation while maintaining regenerative effects. Key pathways, including NF-κB, C/EBPβ, and cGAS-STING, are discussed alongside pharmacological, immunotherapeutic, gene-editing, and epigenetic interventions. SASP heterogeneity necessitates tissue-specific biomarkers for personalized therapies. Challenges include immune interactions, long-term safety, and ethical considerations. SASP modulation emerges as a promising strategy for aging, oncology, and tissue repair, with future advancements relying on multi-omics and AI-driven insights to optimize clinical outcomes.

## 1. Introduction

**Figure 1:**
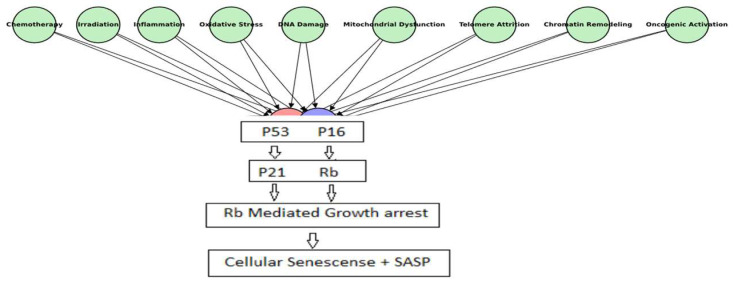
Inducers and pathways of cellular senescence. A schematic overview of major intrinsic and extrinsic inducers of cellular senescence, including oxidative stress, DNA damage, mitochondrial dysfunction, telomere attrition, chromatin remodeling, oncogenic activation, chemotherapy, irradiation, and inflammatory signals. These stressors activate canonical senescence pathways (p53/p21 and p16/Rb), leading to stable cell cycle arrest and the development of the SASP.

Cellular senescence is a complex biological process that leads to stable cell cycle arrest in response to various stressors, playing a critical role in aging, tissue homeostasis, and disease progression [1,2]. Initially described as a replicative limit in human fibroblasts, senescence is now recognized as a multifaceted response triggered by intrinsic and extrinsic factors such as oxidative stress, DNA damage, mitochondrial dysfunction, telomere attrition, chromatin remodeling, oncogenic activation, chemotherapy, irradiation, metabolic stress, inflammation, and altered mechanical cues (Figure 1) [3,4]. These stressors activate key pathways, including the p53/p21 and p16/Rb signaling cascades, leading to stable growth arrest and the development of the SASP [5,6]. While growth arrest remains the defining hallmark of senescence, additional phenotypic changes such as chromatin reorganization, metabolic reprogramming, and morphological alterations also occur [7,8]. The SASP, first described by Coppé et al., 2008, is specifically defined by the secretion of bioactive molecules such as cytokines, chemokines, growth factors, proteases, and extracellular vesicles, which influence the tissue microenvironment [9]. Understanding the regulation of SASP and its impact on cellular function is crucial for developing targeted interventions that modulate senescence for therapeutic benefit. The following sections will explore the physiological and pathological roles of cellular senescence, with a particular focus on SASP as a key mediator of tissue homeostasis and dysfunction.

### 1.1. Cellular Senescence: A Physiological Safeguard and a Pathological Driver

**Figure 2:**
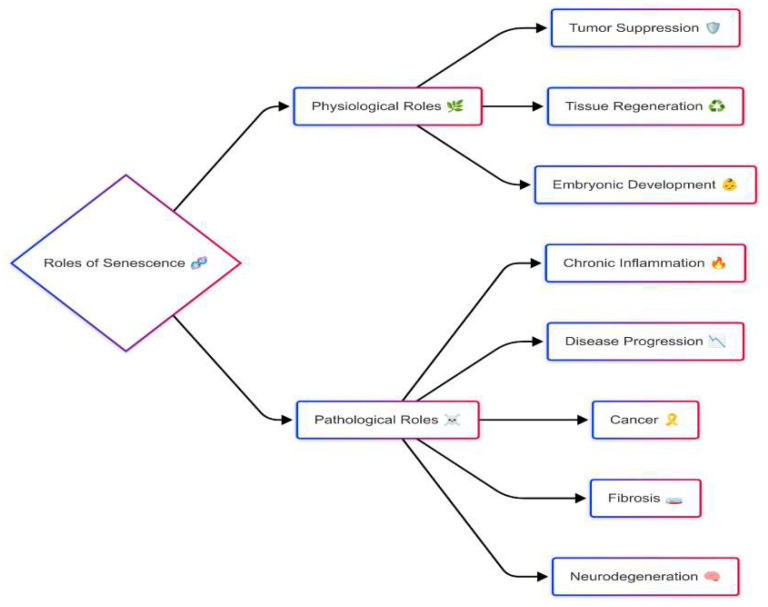
Dual roles of cellular senescence in physiology and pathology. Physiological roles of senescence include tumor suppression, tissue regeneration, and embryonic development. Pathological consequences, particularly when senescent cells persist, include chronic inflammation, disease progression, and promotion of cancer, fibrosis, and neurodegeneration.

Senescence plays a crucial physiological role across various biological processes, contributing to tissue remodeling, wound healing, and embryonic development while serving as a protective mechanism against malignant transformation [10,11] (Figure 2). Senescence halts cell division in response to DNA damage or oncogenic mutations, preventing the spread of faulty genetic material and lowering cancer risk [12]. Additionally, senescent cells accumulate transiently at sites of tissue injury, where their SASP factors modulate immune responses, suppress inflammation, and promote tissue regeneration. During embryonic development, senescent cells help shape tissue structures through morphogenic signaling and are subsequently eliminated via programmed cell clearance to ensure proper organ formation [13]. A defining feature of senescence is the Senescence-Associated Secretory Phenotype (SASP), which comprises a complex mixture of pro-inflammatory cytokines, chemokines, growth factors, and proteases [14]. SASP factors such as interleukin-6 (IL-6), interleukin-8 (IL-8), and tumor necrosis factor-alpha (TNF-α) drive immune cell recruitment and inflammation, while matrix metalloproteinases (MMPs) facilitate tissue remodeling [15]. However, SASP is highly context-dependent, meaning its composition varies based on cell type, tissue environment, and the underlying senescence-inducing stimulus. While these functions are well known, senescence behaves differently in various tissues, making it difficult to define a universal pattern. For instance, senescent fibroblasts in the skin exhibit distinct SASP profiles compared to liver-resident senescent cells [16,17].

This variability complicates therapeutic efforts aimed at targeting senescent cells across diverse tissues [18]. A more refined understanding of tissue-specific senescence may therefore enhance the precision and efficacy of these interventions.

Despite its protective role, persistent senescence can become pathological when senescent cells accumulate beyond their physiological time frame. The prolonged secretion of SASP factors fosters a pro-inflammatory microenvironment that disrupts tissue integrity and function. This chronic, low-grade inflammation, known as inflammaging, contributes to the development of age-associated disorders such as osteoarthritis, atherosclerosis, neurodegenerative diseases, pulmonary fibrosis, and metabolic syndrome (Figure 2) [19,20,21]. Similarly, while cellular senescence suppresses early-stage tumorigenesis, SASP factors can paradoxically promote cancer progression by stimulating angiogenesis, epithelial-to-mesenchymal transition (EMT), and immune evasion [22].

The shift in SASP from tumor suppression to promotion remains poorly understood, with IL-6 and IL-8 playing key roles [23]. This gap in knowledge challenges the effectiveness of senescence-based cancer therapies and highlights the dual role of senescence as protective under normal conditions but pathogenic when dysregulated [24].

### 1.2. SASP as the Mediator of Senescence-Induced Effects

The SASP is the main way in which senescent cells interact with their surroundings. First characterized in 2008, the SASP encompasses cytokines, chemokines, growth factors, proteases, and extracellular vesicles, all of which contribute to immune modulation, tissue remodeling, and inflammatory signaling [9,25]. However, SASP composition changes based on the type of cell, the tissue it originates from, and how long senescence has been active.

SASP’s effects depend on the biological context. In wound healing, SASP factors such as the granulocyte-macrophage colony-stimulating factor (GM-CSF) and CXCL1 recruit immune cells like macrophages and neutrophils to clear senescent cells, promote tissue repair, and restore tissue architecture [4,26]. Conversely, in aging tissues and chronic diseases, persistent SASP activity drives inflammatory signaling, tissue degradation, and cancer progression [27]. For example, tumor necrosis factor-alpha (TNF-α) and matrix metalloproteinases (MMPs) contribute to extracellular matrix degradation and chronic inflammation, exacerbating tissue dysfunction [23]. Additionally, insulin-like growth factor-binding proteins (IGFBPs), particularly IGFBP7, have been implicated in inducing senescence in healthy cells by modulating growth factor signaling pathways, thereby impairing regenerative capacity [28]. While the roles of IL-6, IL-8, and other soluble factors have been well documented, the contribution of extracellular vesicle-derived SASP factors remains less clear [29]. Recent studies have suggested that extracellular vesicles (EVs) may facilitate immune cell recruitment independently of traditional cytokine signaling, indicating the existence of alternative SASP-mediated pathways for tissue modulation [30].

Uncovering the precise functions of these vesicle-mediated interactions could open new avenues for the diagnosis and treatment of age-associated disorders. Developing therapies that can selectively modulate SASP to retain its beneficial effects while suppressing its detrimental outcomes remains a priority for the field.

### 1.3. Cellular Rejuvenation and Tissue Homeostasis: The Therapeutic Potential of SASP Modulation

The interplay between cellular senescence and tissue homeostasis hinges on SASP activity. Cellular rejuvenation, the process of restoring youthful function requires reducing harmful SASP factors while preserving those necessary for tissue repair [31]. Epigenetic reprogramming, mitochondrial restoration, and targeted SASP modulation have emerged as promising strategies for achieving this balance.

Epigenetic reprogramming, particularly through the use of Yamanaka factors (Oct4, Sox2, Klf4, and c-Myc), offers a novel approach to reversing the epigenetic alterations associated with aging. This partial reprogramming does not revert cells to a pluripotent state, but rather it modifies their epigenetic landscape to restore youthful functionality while reducing the pro-inflammatory SASP factors that contribute to cellular senescence. Recent studies have shown that the transient expression of these factors in senescent cells can reduce markers of senescence, modulate SASP components like IL-6 and IL-8, and restore tissue regenerative capacity in aging animal models [32]. This approach holds therapeutic potential by rejuvenating tissues and promoting repair while minimizing harmful inflammation, thereby addressing the adverse effects of SASP in age-related diseases.

In parallel, mitochondrial dysfunction has been identified as a key driver of SASP, and restoring mitochondrial function is critical for suppressing SASP activation. Mitochondrial-targeted therapies, such as antioxidants like MitoQ, have demonstrated the potential to reduce oxidative stress, restore mitochondrial function, and alleviate SASP-driven inflammation in aging tissues. This not only helps improve cellular homeostasis but also enhances tissue repair and regeneration [2].

Senomorphic agents, which specifically target the harmful effects of the SASP without eliminating senescent cells, offer another therapeutic strategy. Compounds like dasatinib and quercetin have shown promise in preclinical models by reducing the pro-inflammatory components of the SASP, thereby improving tissue function and mitigating the chronic inflammation associated with age-related diseases like osteoarthritis and neurodegeneration [33]. These agents promote tissue repair by modulating the SASP, maintaining the beneficial aspects of senescence while reducing its detrimental effects.

Despite the therapeutic promise of these approaches, their clinical translation remains challenging. For example, while rapamycin has shown efficacy in suppressing SASP in preclinical models, its long-term use could impair immune surveillance, especially in cancer-prone tissues, potentially accelerating tumor progression [34]. This highlights the necessity for context-dependent SASP modulation, where the specific tissue environment and senescence burden must be considered to avoid unintended side effects.

The SASP influences stem cell function, immune responses, and extracellular matrix balance, making it a target for enhancing tissue repair and reducing chronic inflammation [35,36]. Despite its therapeutic potential in aging and disease, the SASP’s tissue-specific variability poses a major challenge. This review critically explores the recent advances in understanding the SASP’s role in aging, pathology, and regenerative therapies.

## 2. SASP: The Molecular Nexus of Cellular Senescence

The SASP is a crucial interface through which senescent cells influence their environment by secreting cytokines, chemokines, growth factors, and extracellular vesicles, affecting tissue remodeling and immune responses [2,31]. Once seen as a passive byproduct, the SASP is now recognized for its active, context-dependent roles. Modulating the SASP, reducing its inflammatory components while retaining regenerative effects, offers therapeutic potential for tissue rejuvenation and homeostasis [37], necessitating deeper insights into its composition, dynamics, and tissue-specific actions.

### 2.1. Molecular Composition of SASP: Beyond the Core Components

The SASP secretome comprises a diverse array of soluble and insoluble factors, including pro-inflammatory cytokines (e.g., IL-6, IL-8, TNF-α), chemokines (e.g., CCL2, CXCL1), growth factors (e.g., VEGF, HGF), matrix metalloproteinases (MMPs) (e.g., MMP-1, MMP-3), and extracellular vesicles containing proteins, lipids, and regulatory RNAs [5,38]. The secretion of these factors is predominantly governed by the NF-κB, C/EBPβ, and p38 MAPK signaling pathways, all of which respond to cellular stress and DNA damage signals [39,40,41].

Although SASP profiling has advanced, the roles of many components such as extracellular vesicle-derived microRNAs (EV-miRNAs) in immune modulation remain unclear due to methodological inconsistencies [42]. SASP heterogeneity, influenced by senescence triggers and tissue context, complicates biomarker development [43,44]. Still, this diversity offers therapeutic opportunities; the selective suppression of pro-inflammatory SASP elements while preserving regenerative factors may enhance tissue homeostasis without immunosuppression [23].

### 2.2. Temporal Dynamics of SASP: A Shifting Molecular Landscape

The SASP evolves and is initially dominated by pro-inflammatory cytokines and chemokines, which recruit immune cells such as macrophages and NK (natural killer) cells [45,46]. In later stages, it shifts toward an ECM (extracellular matrix)-focused profile enriched with MMPs (matrix metalloproteinases) and TIMPs (tissue inhibitors of metalloproteinases), contributing to fibrosis and chronic inflammation [47,48,49]. ATM (ataxia-telangiectasia mutated) and ATR (ataxia-telangiectasia and Rad3-related) kinases regulate this transition, although the precise mechanisms remain unclear [50,51]. Understanding these time-dependent changes is key for targeted therapies, as early SASP inhibition may impair repair [35], while late suppression could reduce fibrosis in diseases like osteoarthritis and atherosclerosis [49]. The subsequent table delineates the principal distinctions between early and late SASP [Table 1].

### 2.3. Tissue-Specific Variability: SASP Across Different Microenvironments

**Figure 3:**
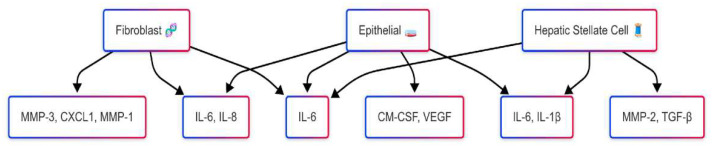
SASP variability across cell types. This schematic illustration highlights the heterogeneity of SASP components secreted by different senescent cell types, including fibroblasts, epithelial cells, and hepatic stellate cells. While certain factors such as IL-6 are commonly expressed across all three cell types, others like MMP-1 (fibroblasts), VEGF (epithelial cells), and TGF-β (hepatic stellate cells) exhibit cell-type specificity. This variability reflects the context-dependent roles of the SASP in tissue remodeling, inflammation, and disease progression.

SASP composition is highly tissue-specific, with profiles from hepatic stellate cells differing significantly from dermal fibroblasts, influenced by cellular origin and microenvironment [52,53]. This variability poses diagnostic challenges in defining universal biomarkers but offers opportunities for tissue-targeted therapies. In liver fibrosis, for example, hepatocyte-specific SASP modulation via targeted delivery systems has shown promise [54]. However, reliance on fibroblast-based models limits generalizability, as senescence differs across epithelial, endothelial, and immune cells [43,55]. Future research should prioritize organ-specific models for clinical relevance. A comparative analysis illustrates the SASP variability among fibroblasts, epithelial cells, and hepatic stellate cells [9,56] (Figure 3).

### 2.4. SASP Signaling Pathways

SASP production is primarily regulated by the NF-κB and C/EBPβ pathways, which are activated by cellular stress and DNA damage [9,41]. While these pathways are central to SASP induction, the signals driving cell-type-specific variations remain unclear. Emerging evidence suggests that lncRNAs (long non-coding RNAs) can modulate SASP components independently of cell cycle arrest [5]. Therapeutically, RNA-targeted strategies show promise, although systemic NF-κB inhibition may impair immune function and elevate cancer risk.

### 2.5. SASP and the Immune System: Friend or Foe?

The immune system regulates the SASP effects by clearing senescent cells through macrophages, NK cells, and T lymphocytes, maintaining tissue homeostasis and preventing tumorigenesis [57,58]. With aging, impaired immunity allows SASP accumulation, driving chronic inflammation. In cancer, the SASP initially aids immune-mediated tumor suppression but later promotes tumor growth via immunosuppressive cytokines [59]. Strategies like SASP-targeting vaccines and immune checkpoint inhibitors aim to restore surveillance without inducing immune exhaustion [58]. Given its dynamic nature, SASP modulation offers opportunities to balance inflammation control and tissue repair for therapeutic rejuvenation.

## 3. Cellular Rejuvenation Through SASP Modulation

Cellular rejuvenation involves restoring function in aged or damaged cells [31]. While senescence aids in tumor suppression and repair, persistent senescent cells and a sustained SASP can drive tissue dysfunction through chronic inflammation and matrix degradation [60,61]. Modulating the SASP to suppress harmful factors while retaining regenerative ones is a promising strategy for improving tissue health [62]. However, the dynamic, context-dependent nature of the SASP raises concerns about long-term effects, including the potential disruption of immune function and regeneration [63]. This provides a foundation for exploring therapeutic strategies that modulate the SASP to achieve rejuvenation outcomes.

One such approach is epigenetic reprogramming, which targets DNA methylation, histone modifications, and chromatin structure to restore youthful cellular function without altering cell identity. The transient use of Yamanaka factors Oct4, Sox2, Klf4, and c-Myc has demonstrated potential in reversing age-related epigenetic changes without inducing full reprogramming [64]. Despite its promise, this strategy raises safety concerns, as these factors can induce double-strand DNA breaks, compromising genomic integrity in some cell types [32]. To address these risks, recent efforts have focused on small-molecule interventions that modulate epigenetic enzymes such as histone deacetylases (HDACs) and DNA methyltransferases (DNMTs) [65]. These compounds offer more controlled alternatives, although off-target effects remain a concern. For instance, HDAC inhibitors, while therapeutically promising, can influence multiple cellular pathways, potentially leading to toxicity in non-target tissues [66,67]. Therefore, optimizing treatment protocols remains essential for balancing rejuvenation with genomic stability.

In parallel, pharmacological strategies specifically targeting SASP have advanced through the development of senolytic and senomorphic therapies. Senolytics such as dasatinib, quercetin, and navitoclax eliminate senescent cells by targeting pro-survival proteins like BCL-2, BCL-XL, and MCL-1, thereby reducing SASP at its source and improving tissue function in preclinical models [68,69]. However, off-target effects, such as navitoclax-induced thrombocytopenia, highlight the need for more tissue-specific approaches [70]. Senomorphics, including rapamycin and metformin, suppress the SASP via the mTOR and NF-κB pathways while preserving the regenerative functions of senescent cells [71]. Nonetheless, excessive suppression can impair physiological functions like wound healing and immune recruitment [36,72], underscoring the importance of distinguishing pathological from beneficial SASP activity.

Mitochondrial health is another critical regulator of SASP and cellular aging. Mitochondrial dysfunction contributes to SASP through metabolic reprogramming, increased ROS, and impaired mitophagy. It also activates the cGAS-STING pathway by detecting mitochondrial DNA (mtDNA) released during dysfunction, triggering pro-inflammatory cytokine secretion [73]. In this context, interventions that restore mitochondrial function, such as NAD^+^ precursors like nicotinamide riboside (NR) or mitophagy inducers like urolithin A, have shown potential to suppress SASP and improve cellular health [74,75]. However, their long-term impact and optimal dosing remain to be defined, especially given the potential for chronic mitochondrial activation to induce hypermetabolic stress.

Given SASP’s strong immunomodulatory role, immune system function is tightly linked to the success of SASP-targeted therapies. Under normal conditions, SASP factors attract immune cells such as macrophages, NK cells, and CD8^+^ T cells to clear senescent cells and support tissue repair. With aging, however, this immune clearance declines, allowing senescent cells to accumulate and drive chronic inflammation [76]. Immunomodulatory strategies, including PD-1/PD-L1 checkpoint inhibitors and SASP-targeted vaccines, aim to restore immune surveillance without impairing tissue regeneration [77]. The key challenge is to activate immune responses against senescent cells without provoking autoimmunity or damaging healthy tissue.

To refine these immunotherapeutic approaches, recent drug development has focused on precision-targeting SASP regulatory pathways. Small molecules and biologics, such as JAK inhibitors, have demonstrated the ability to suppress pro-inflammatory SASP by blocking IL-6/STAT3 signaling [78]. However, broad-spectrum inhibitors like JAK and NF-κB carry the risk of disrupting SASP’s beneficial roles in tissue-specific immune repair [6,79]. Consequently, future therapies must selectively inhibit harmful SASP components while preserving those essential for tissue maintenance.

Altogether, SASP modulation offers a compelling avenue for promoting cellular rejuvenation, enhancing tissue repair, and mitigating age-related inflammation. Targeting upstream pathways including epigenetic regulators, mitochondrial dynamics, and immune-mediated signaling may restore tissue homeostasis and support healthy aging [80]. Yet, the heterogeneity of SASP across cell types and tissues reinforces the need for precision therapies that balance efficacy with safety to achieve optimal therapeutic outcomes.

## 4. SASP and Its Impact on Tissue Homeostasis

The SASP influences tissue homeostasis by secreting cytokines, chemokines, growth factors, and matrix-modifying enzymes that regulate tissue remodeling, immune responses, and stem cell niche function. Its effects are highly context-dependent, varying with tissue type, environment, and duration of activity [81]. While a transient SASP supports repair and regeneration, persistent secretion leads to chronic inflammation, structural disruption, and age-related disease [2]. This dual role is evident in contrasting outcomes beneficial during embryonic development and wound healing, yet harmful in degenerative aging tissues [76].

This context-dependent behavior is particularly evident in tissue remodeling and regeneration. SASP factors are essential during wound healing, with senescent cells secreting matrix metalloproteinases (MMPs) and growth factors that degrade damaged extracellular matrix (ECM) and promote regeneration through immune cell recruitment and angiogenesis [82,83]. While beneficial in acute repair, a prolonged SASP—such as sustained MMP-2 and MMP-9 secretion—can lead to pathological fibrosis, as seen in pulmonary fibrosis [84]. Therapeutically, modulating the SASP by inhibiting pro-fibrotic signals like TGF-β and MMP-9 may preserve ECM balance while retaining regenerative functions [85].

In parallel, SASP-mediated inflammation plays a dual role in tissue homeostasis. It supports tissue repair by promoting immune cell recruitment through pro-inflammatory factors such as IL-6, IL-8, and TNF-α [86]. However, when dysregulated with aging, SASP leads to chronic low-grade inflammation, or inflammaging, impairing immune function and tissue repair [87]. This prolonged inflammatory state is maintained by the continued activation of pattern recognition receptors (PRRs) and the cGAS-STING pathway, even after senescent cells are cleared [88]. The resulting inflammaging contributes to age-related diseases like atherosclerosis and Alzheimer’s. Although JAK inhibitors can suppress SASP activity, their broad immunosuppressive effects highlight the importance of selectively modulating SASP-driven inflammation [89].

Beyond immune responses, SASP significantly influences stem cell niche dynamics. Stem cell niches rely on tightly regulated signaling for self-renewal and differentiation. SASP factors such as IL-6, IL-8, and CCL2 modulate the niche environment, supporting regeneration during acute injury but impairing stem cell function when chronically expressed [42,90]. In hematopoietic stem cells (HSCs), prolonged IL-6 exposure leads to myeloid skewing and diminished lymphoid output, weakening hematopoiesis and immune competence in aging individuals [91]. It has been hypothesized that the SASP may act as an adaptive mechanism to suppress stem cell activity and reduce oncogenic risk, though this concept requires further empirical validation [90]. Understanding the SASP’s influence on stem cell maintenance across diverse tissue environments is essential for designing effective rejuvenation therapies.

When unregulated, persistent SASP activity contributes to tissue degeneration through chronic inflammation, ECM degradation, and fibrotic remodeling marked by excessive collagen deposition and reduced elasticity [92]. In conditions such as osteoarthritis and pulmonary fibrosis, SASP-activated fibroblasts drive abnormal collagen accumulation and reduce ECM turnover, even in the absence of inflammatory cytokines [93]. These findings suggest SASP can influence fibroblast behavior through non-inflammatory mechanisms. Current therapeutic strategies include fibroblast-targeted inhibitors and temporally controlled interventions that confine SASP activity to early injury phases, supporting regeneration while minimizing the risk of chronic fibrosis [23,94].

Altogether, the SASP represents a central regulatory mechanism in tissue homeostasis. Its functional transition from reparative to degenerative with age presents a key challenge for therapeutic design [95]. Targeted strategies that tailor SASP modulation to specific tissue contexts and cellular states hold promise for preserving tissue integrity while mitigating age-related dysfunction [87,95]. A deeper understanding of the molecular mechanisms driving SASP-induced tissue changes will be crucial for developing precise and safe therapies aimed at restoring tissue health.

## 5. Emerging Therapeutic Strategies for SASP Modulation

The modulation of the SASP has emerged as a promising strategy for managing aging, cancer, and age-related diseases by either suppressing harmful inflammatory signals or eliminating senescent cells. While preclinical studies are encouraging, clinical translation is challenged by the heterogeneous and context-dependent nature of SASP across tissues [96]. Human trials often yield variable outcomes due to differences in SASP composition, immune responses, and repair mechanisms [97]. The long-term effects of these therapies on immune function and tissue integrity remain unclear. Ongoing research explores advances in pharmacological, immunotherapeutic, gene-based, and epigenetic approaches, along with novel delivery systems.

Among these, pharmacological strategies remain at the forefront, including senolytics, which induce apoptosis in senescent cells, and senomorphics, which suppress SASP secretion without affecting cell viability. The dasatinib–quercetin combination has shown efficacy in various tissues, while navitoclax, a Bcl-2 family inhibitor, effectively targets senescent lung fibroblasts and bone marrow stromal cells [61,86]. However, concerns remain over off-target effects, such as navitoclax-induced thrombocytopenia [69], and the limited expression of markers like p16^INK4a across senescent populations, which complicates specificity and therapeutic effectiveness [98]. Senomorphic therapies act by inhibiting SASP production through key signaling pathways such as mTOR, NF-κB, and JAK/STAT. Agents like rapamycin, metformin, and JAK inhibitors have demonstrated efficacy, with rapamycin shown to reduce IL-6 and IL-8 while preserving the beneficial SASP components involved in tissue repair [71,99,100]. Additionally, emerging evidence points to the regulatory role of non-coding RNAs, particularly microRNA-mediated feedback loops, in fine-tuning SASP activity [101,102]. Despite their promise, RNA-targeting strategies are limited by tissue specificity and the potential for disrupting essential regulatory networks.

Complementing pharmacological efforts, immunotherapeutic approaches seek to enhance the immune system’s role in regulating SASP activity. SASP factors such as IL-6, CXCL1, and CCL2 recruit immune cells including NK cells, macrophages, and T lymphocytes to clear senescent cells and maintain tissue integrity [6,57]. Strategies involving immune checkpoint inhibitors such as anti-PD-1 and anti-CTLA-4 antibodies have shown potential to restore senescence surveillance. However, senescence-associated PD-L1 upregulation may suppress T-cell activity, raising concerns about immune tolerance and autoimmunity [91,103,104]. Identifying biomarkers that can distinguish pathological SASP-driven immunosuppression from physiological tolerance is crucial for optimizing the safety of these interventions. In parallel, SASP-targeted vaccines have emerged as a novel approach to stimulate immune responses against senescent cell antigens. A peptide-based vaccine has demonstrated reductions in SASP-driven inflammation and improved tissue repair in aged mouse models [105]. Yet, translating these findings to humans presents challenges such as immune exhaustion in older adults and variability in SASP-associated surface markers across tissues [106].

Beyond immunotherapy, gene therapy offers a precise strategy to modulate the SASP at the genetic level. Tools like CRISPR-Cas9, RNA interference (RNAi), and epigenetic editing have been used to silence key SASP genes such as IL-6, IL-8, and CXCL1 [107]. For example, RNAi-mediated knockdown of IL-6 has successfully reduced inflammatory cytokine secretion while preserving senescence-associated growth factors in preclinical studies [108]. Despite this promise, clinical application is limited by delivery barriers, immune responses, and off-target effects. To overcome these challenges, tissue-specific promoters such as hepatocyte-selective elements have been proposed to restrict gene editing to target organs, reducing systemic impact [109]. Continued improvements in vector design, immunogenicity, and therapeutic durability will be critical for the success of gene-based SASP modulation.

In parallel, epigenetic reprogramming represents a complementary approach by reversing age-associated gene expression patterns that contribute to the SASP. Epigenetic mechanisms including DNA methylation, chromatin remodeling, and histone acetylation govern the transcriptional regulation of SASP genes [110]. The transient expression of Yamanaka factors (Oct4, Sox2, Klf4, and c-Myc) has shown the ability to restore youthful gene profiles in senescent cells without triggering full pluripotency [64]. However, this approach carries risks, particularly genomic instability in aged tissues with compromised DNA repair capacity. As a pharmacological alternative, histone deacetylase inhibitors (HDACis) have shown promise in downregulating pro-inflammatory genes and enhancing cellular plasticity [111]. Yet, their broad activity introduces concerns regarding off-target effects and epigenetic drift, necessitating further refinement for safe and targeted application.

Equally important to therapeutic success is the development of effective drug delivery systems. Traditional delivery methods often struggle with low bioavailability and insufficient tissue penetration, particularly when targeting senescent cells in complex tissue environments. Nanoparticles have emerged as promising vehicles for senolytic and senomorphic agents. Engineered with ligands targeting markers like SA-β-galactosidase or p16^INK4a, these nanoparticles enhance drug localization while minimizing systemic toxicity [112,113]. Nonetheless, rapid clearance by the reticuloendothelial system (RES), especially in the liver, poses a major limitation. Surface modifications such as PEGylation have improved circulation time, but achieving reliable, tissue-specific delivery remains a central challenge [114].

## 6. Two Paths to Cellular Rejuvenation: Senescence Clearance vs. SASP Modulation

The growing recognition of cellular senescence as a contributor to age-related diseases has driven interest in two main therapeutic strategies—senescence clearance and SASP modulation. Both aim to restore tissue homeostasis and reduce chronic inflammation but differ in mechanism, therapeutic reach, and long-term effects [115]. The choice between them is context-dependent senescent cell clearance may impair tissue repair where transient senescence is beneficial, while SASP modulation alone may inadequately address chronic inflammatory damage [81,116]. Emerging research increasingly supports the integration of both strategies to optimize cellular rejuvenation outcomes.

Mechanistically, senescence clearance and SASP modulation differ in both target and mode of action. Clearance eliminates senescent cells using senolytics, immune responses, or genetic ablation targeting markers like p16^INK4a and p21^CIP1, thereby reducing inflammation and restoring tissue function [116]. In contrast, SASP modulation alters the secretory profile of senescent cells while preserving their viability. Senomorphic agents such as JAK inhibitors and rapamycin suppress pro-inflammatory SASP components while retaining the regenerative signals essential for tissue maintenance [100]. These distinctions have significant therapeutic implications; while clearance removes the inflammatory source, it may also eliminate reparative signaling. SASP modulation preserves these regenerative functions but could risk immune evasion, particularly in tumor-prone environments.

Preclinical research has further clarified the relative efficacy of these strategies across different tissues. Senolytics like the dasatinib–quercetin (D + Q) combination have shown promise in selectively eliminating senescent cells, thereby improving tissue function and extending lifespan. In aged mice, the D + Q treatment enhanced muscle strength, endurance, and mitigated age-related intervertebral disc degeneration, preserving tissue integrity [36,86,117]. Conversely, SASP modulation has demonstrated value in neurodegenerative models, where rapamycin effectively reduced neuroinflammation and preserved cognitive function by attenuating SASP-driven cytokine production [118]. These findings highlight the tissue-specific nature of therapeutic responses. Senescence clearance appears more effective in musculoskeletal tissues where senescent cells impair regeneration, while SASP modulation may be more suitable for the brain, where inflammatory signaling plays a greater role than structural remodeling.

While both strategies hold promise, they also pose distinct safety challenges. Senolytics such as navitoclax are effective in eliminating senescent cells but carry risks of off-target cytotoxicity, including thrombocytopenia due to platelet depletion [69]. Moreover, indiscriminate clearance may inadvertently remove beneficial transient senescent cells that contribute to wound healing, impairing tissue repair [119]. In comparison, SASP modulation offers greater tissue specificity by targeting signaling pathways like mTOR and JAK/STAT without inducing cell death. However, the prolonged suppression of key cytokines such as IL-6 and IL-8 could compromise immune function and tissue repair capacity and may even increase vulnerability to tumor development due to impaired immune surveillance [57,120].

These safety considerations further underscore the importance of context in selecting therapeutic strategies. In musculoskeletal tissues like cartilage and skeletal muscle, senescence clearance effectively restores regeneration by removing pro-inflammatory SASP-secreting cells [121,122]. By contrast, SASP modulation may be better suited for neural tissues, where the post-mitotic nature of neurons and supportive roles of glial-derived SASP factors help maintain synaptic plasticity and function [123].

In cancer therapies, modulation of the SASP can unintentionally promote tumor progression by facilitating immune evasion. This occurs through the action of cytokines such as the pro-inflammatory IL-6, which contributes to chronic inflammation and indirectly promotes immune suppression, and IL-10, which exerts direct immunosuppressive effects. In such scenarios, the clearance of senescent cells may be preferable to eliminate pro-tumorigenic influences [6,124]. In contrast, in repair-prone tissues, SASP modulation often proves advantageous by preserving growth factors like VEGF and HGF that are essential for regeneration while limiting chronic inflammation [23,36]. Collectively, these findings emphasize the need for precision therapies tailored to specific senescence profile strategies that can suppress pathological SASP activity without disrupting critical homeostatic functions.

To address the limitations of single-modality approaches, emerging research supports combining senescence clearance and SASP modulation into hybrid therapeutic protocols. These dual strategies aim to harness the strengths of both methods while mitigating their respective risks. For instance, senolytics like dasatinib and quercetin have demonstrated benefits in clearing senescent cells and improving physical function, while SASP modulators such as rapamycin reduce inflammatory cytokine output and preserve cognitive performance in aged murine models [86]. A proposed dual approach involves initially modulating SASP to reduce inflammation, followed by senolytic treatment to eliminate residual senescent cells. Although this sequential strategy has yet to be validated in direct preclinical studies, similar “one–two punch” models have been tested in oncology, where pro-senescence therapies are followed by senolytics to enhance cancer cell elimination. This paradigm leverages the vulnerabilities of senescent tumor cells and offers a framework that could be adapted for regenerative medicine [36]. Future studies should focus on optimizing the timing, dosage, and sequencing of combined strategies to maximize therapeutic synergy and minimize risk, thereby advancing the clinical translation of senescence-targeting therapies for age-related disorders and tissue rejuvenation.

## 7. Challenges and Future Directions

Modulation of the SASP has gained attention as a therapeutic strategy for combating age-related diseases, tissue degeneration, and cancer progression. While preclinical studies show promise, clinical translation remains limited due to the heterogeneous and context-specific nature of SASP, as well as its complex crosstalk with immune pathways [56]. Addressing these challenges requires integrated efforts in molecular biology, pharmacology, and computational sciences to develop targeted, tissue-specific therapies.

The SASP is not a uniform signature but varies depending on cell type, senescence trigger, tissue environment, and duration. While core components like IL-6, IL-8, and CXCL1 are commonly expressed, others such as extracellular vesicle-derived microRNAs and long non-coding RNAs show high tissue specificity [23,101]. This molecular diversity complicates biomarker discovery and universal therapy design. Advances in single-cell RNA sequencing and spatial transcriptomics have enhanced our understanding of SASP heterogeneity, although technical limitations persist [125]. Machine learning tools capable of integrating multi-omic datasets may help create personalized approaches for SASP modulation [126].

Therapeutically, SASP displays both beneficial and detrimental roles depending on context. Acute SASP promotes regeneration, wound healing, and embryonic development, but chronic SASP contributes to inflammaging, fibrosis, and cancer [21]. For instance, senescent fibroblasts secrete pro-angiogenic factors, aiding repair while also facilitating tumor growth and immune evasion in epithelial tissues [127]. Mitochondrial dysfunction, particularly via the cGAS-STING pathway, may drive chronic SASP and associated inflammation [88], yet targeting mitochondria raises concerns over the long-term effects on metabolic integrity [75].

The immune system is both influenced by and responsive to the SASP. Early SASP supports immune recruitment through cytokines like IL-6 and CXCL2, promoting senescent cell clearance [128,129]. However, a persistent SASP can drive immune exhaustion and chronic inflammation, suppressing anti-tumor responses through elevated levels of IL-6 and TGF-β [59,129]. Immunotherapies such as PD-1/PD-L1 inhibitors offer partial success but require a deeper understanding of SASP-immune dynamics to improve consistency and efficacy [61,78].

Translating preclinical findings into clinical applications presents further obstacles. Murine models often fail to replicate human senescence biology due to species-specific differences in SASP and immune responses [61,130]. Emerging platforms such as humanized organoid systems and patient-derived senografts offer better fidelity but are hampered by inconsistent induction methods and limited standardization. Collaborative research frameworks and harmonized protocols will be essential for achieving reproducible clinical outcomes.

In parallel, the ethical and societal implications of SASP-targeting therapies are coming to the forefront. While aimed at treating age-related diseases, these therapies raise concerns over accessibility, equitable use, and potential application in cosmetic or non-medical contexts. The long-term societal impact of extending healthspan also warrants consideration, including effects on retirement and healthcare systems. Regulatory groups such as the International Society on Aging and Longevity advocate for global ethical standards, emphasizing safety, equitable access, public engagement, and well-defined policies [131].

SASP modulation represents a promising area of research in regenerative medicine and aging. However, to realize its full potential, continued progress is needed across several fronts.

Key priorities for future research include:

- Identifying tissue-specific biomarkers to track SASP dynamics in health and disease;
- Developing next-generation senolytics and senomorphics with greater specificity and improved safety profiles;
- Refining organoid models to better mimic human tissue responses to SASP-targeting interventions;
- Implementing long-term studies to assess the safety and efficacy of SASP modulation therapies.

The integration of cutting-edge technologies, such as AI-driven drug discovery and multi-omics profiling, will be essential for advancing SASP modulation and fully unlocking its therapeutic potential.

## 8. Conclusions

Cellular senescence, once seen as an irreversible process, is now recognized as a dynamic phenomenon with critical roles in tissue homeostasis, aging, and disease. At its core lies the SASP, a tissue-specific secretome with both beneficial and detrimental effects. This review highlights the SASP’s molecular mechanisms, physiological roles, pathological implications, and therapeutic modulation strategies for cellular rejuvenation. While a transient SASP supports tissue repair and immune surveillance, a persistent SASP drives chronic inflammation, fibrosis, and cancer. Therapeutic strategies focus on senescence clearance removing senescent cells to reduce the SASP at the cost of wound-healing cells or SASP modulation, which selectively alters secretory profiles to suppress inflammation while preserving reparative signals, although with potential immune suppression risks.

A key challenge is the SASP’s tissue-specific heterogeneity, influenced by cell type, senescence triggers, and microenvironment. Senescence clearance is more effective in muscle and cartilage, where senescence hinders regeneration, while SASP modulation benefits neurodegenerative contexts by mitigating inflammation without depleting supportive glial cells. Moving forward, tissue-specific biomarkers, optimized therapeutic protocols, and technologies like single-cell sequencing and machine learning will enhance the precision and safety of SASP-targeting interventions. Modulating SASP represents a promising approach for extending health span and improving quality of life through targeted molecular therapies.

## Figures and Tables

**Table 1:** Early SASP (pro-repair) versus late SASP (pro-degradation).

Feature	Early SASP (Pro-Repair)	Late SASP (Pro-Degradation)
Key factors	Growth factors, anti-inflammatory cytokines, matrix remodeling proteins	Pro-inflammatory cytokines (IL-6,8), chemokines, matrix metalloproteins (MMPs)
Function	Facilitates tissue repair and regeneration	Promote tissue degradation and chronic inflammation
Temporal dynamics	Occurs in the initial stages of senescence, transient and resolves upon completion of repair	Develops during prolonged senescence, is persistent, and can contribute to age-related pathologies
Impact on environment	Supports regenerative processes and maintains tissue homeostasis	Disrupts tissue homeostasis, promotes inflammatory factors; may lead to tumor progression or fibrosis depending on the context

## Data Availability

Not applicable.
